# Is It Time to Re-Shift the Research Agenda? A Scoping Review of Participation Rates in Workplace Health Promotion Programs

**DOI:** 10.3390/ijerph20032757

**Published:** 2023-02-03

**Authors:** Katarina Bensa, Klemen Širok

**Affiliations:** 1 Faculty of Management, University of Primorska, 6000 Koper, Slovenia; 2 Faculty of Health Sciences, University of Primorska, 6310 Izola, Slovenia

**Keywords:** workplace health promotion program (WHPP), participation rate, worksite, employee, scoping review

## Abstract

Workplace health promotion programmes (WHPPs) are among the most important measures to improve the health and motivation of the ageing workforce. However, they are accompanied with certain challenges, such as low participation rates and higher participation levels of the more health-conscious workers, often failing to engage those who need such interventions the most. Following the PRISMA guidelines, this scoping review examined participation rates reported in articles on WHPPs to identify potential knowledge gaps. The results are worrying: participation rates are not only infrequently reported, but also low. Of the 58 articles, 37 report participation rates, with the majority (20) reporting an average participation rate of less than 50%. Reported participation rates refer either to different target groups, the type of intervention, or to single points in time, which makes it difficult to establish consistent criteria for comparison. We argue that despite the importance of WHPP efficacy, research focus should shift to the determinants of participation, as well as the issue of standardising the reporting of participation rates, alongside the potential problem of reporting bias.

## 1. Introduction

According to the OECD data on retirement age, the duration of working life in most OECD countries has increased since 2000 [1] due to specific work patterns and demographic changes. The proportion of older workers has increased as well. Workplace health promotion programmes (WHPPs) are among the most important measures to improve the health and motivation of the ageing workforce, as well as the working conditions themselves. Thus, choosing an effective WHPP has a positive impact on maintaining and strengthening workers’ physical [2] and mental health [3]. It contributes to actual vitality and work engagement and has the potential to prolong working life [2,4]. Workplace health promotion is necessary not only for employees but also for employers, as it brings many benefits to companies, such as improved work performance [5,6], higher work engagement [5], better work ability, higher job retention rate [6], and a lower financial burden due to a reduction in sick leave [7]. Thus, WHPPs can be profitable. An analysis of 51 studies with 261,901 participants and 122,242 controls from nine economic sectors in 12 countries, published between 1984 and 2012, found that overall weighted return on investment (ROI) yielded a total of USD 2.38 for every dollar invested [8].

However, WHPPs come with certain challenges and the reality is far from perfect. Studies suggest that participation rates in such programmes are often below 50 per cent [9] and typically range between 20 and 30 per cent [10]. Moreover, healthier and more health-conscious workers predominate among WHPP participants [11,12]. On the other hand, those in need of such interventions often fail to participate or to be activated by these programmes in the first place [13]. There is some limited evidence that preventive interventions or programmes, while effective, also have deadweight effects [14,15], the extent to which the (expected) effects would have occurred even without the intervention. This means that some participants in these programmes are already health conscious in the first place and are thus rewarded for performing something or being offered something for free that they would have implemented anyway. This significantly reduces the potential benefits and cost-effectiveness of these programmes [10,16].

Although 50 to 75 per cent of workers do not choose to participate in WHPPs, the field of employees’ commitment to personal health is still fairly unexplored. Some of these challenges have been known for a long time, with the 2013 research agenda published in the American Journal of Health Promotion (AJHP) calling for more research to focus on organisational culture, incentives, and social marketing in WHPPs to address uptake issues [17]. The question, however, is just how much the research focus has actually shifted over the past 10 years. The authors fear not much. First, our literature search revealed that no review articles had examined WHPP participation rates. Second, a brief review of abstracts in the AJHP showed that such issues were addressed in only a few articles. Third, WHPP participation rates are (mostly, as we found in our scoping review) still below 50%.

### Research Problem and Aim

WHPP researchers and practitioners seem to forget that the problem with the overall effectiveness of WHPPs is not the effects of WHPPs as such, but rather the low participation rates to begin with. Thus, the research field may have a problem that we are not even aware of. There is a large body of research in the field of WHPPs that focuses only on individual interventions and programmes and their effects. This calls into question its usefulness, given that the real problem appears to be low participation.

The only systematic review of participation rates we came across is Ryde et al., (2013) [18], which covers research up to 2010. However, it only considers workplace physical activity programmes and describes the characteristics of studies with high participation rates. The aim of our scoping review is more ambitious. First, our scoping review covers all articles up to April 2022, whereas the mentioned review only covers research from 1977 to 2010. Second, by including all WHPPs, we do not limit ourselves to physical activity programmes. Third, our analysis considers WHPP specifics that (on face value) relate to participation rates.

In this article, we present a scoping review of WHPP participation rates reported in research using the PRISMA model [19]. To shed light on this particular research topic, the analysis also considers some characteristics of WHPPs and organisations that seem to be related to WHPP participation rates. In addition to the level of employee participation, the analysis also takes into consideration the type of programme or intervention, the target group(s) of the intervention, the type of organisation where the intervention took place, and the methods used to recruit employees to participate in the intervention. 

The article is structured as follows. The following section presents the application of the PRISMA method. The methods section is followed by the analysis of the results. Next, we discuss the results and point out the limitations of our scoping review. The article concludes with suggestions for future research and implications for the WHPP field.

## 2. Methods

### 2.1. Search Strategies

The scoping review was based on a literature search in bibliographic databases, namely PubMed, SAGE Journals, JSTOR, and Emerald in 2021–2022. In addition, a manual search of Google Scholar was conducted to identify articles that may have been missed in the database search. The search used a combination of terms and keywords, which were adjusted and refined during the search to yield more appropriate results. The first search used a combination of terms (workplace OR worksite OR employee*) AND (health promotion OR well-being OR well-being OR wellness) AND (program* OR intervention) AND (participation) AND (motives OR factors OR determinants). As the results were not entirely suitable, we adjusted the search terms and used a new combination of terms in the second search: (workplace OR worksite OR employee*) AND (health promotion OR well-being OR well-being OR wellness) AND (program* OR intervention) AND (participation rate OR recruitment OR engagement OR attendance OR “take-up”) AND (rate). In the third search iteration, the term “take-up rate” was excluded from the search. An obstacle arose when searching the JSTOR article database, where the length and number of search terms and keywords are limited, which meant that the search terms had to be adapted again: (workplace OR worksite OR employee*) AND (health promotion OR wellbeing OR wellness) AND (program* OR intervention) AND (participation OR recruitment OR engagement) AND (rate).

### 2.2. Inclusion and Exclusion Criteria

**Figure 1:**
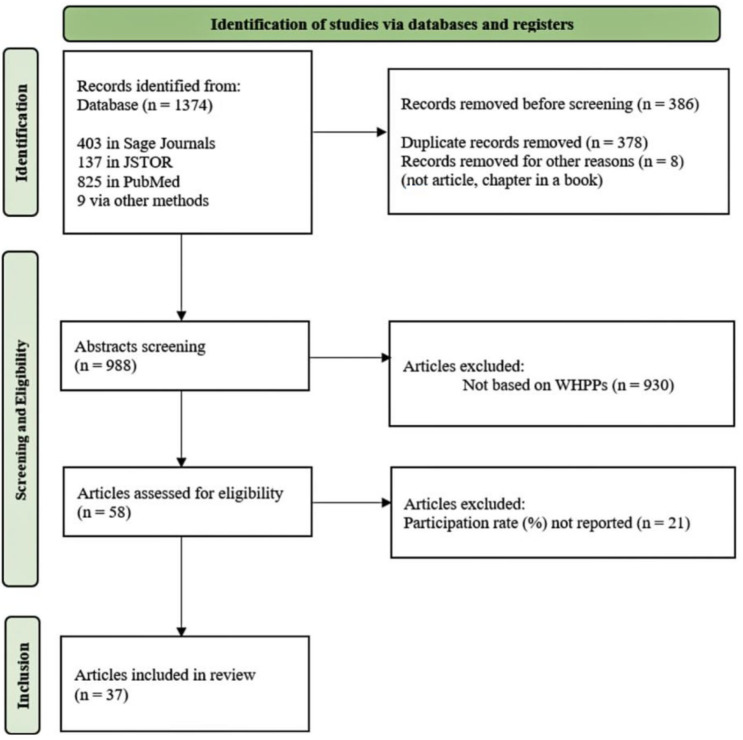
PRISMA model; identification of studies for the scoping review.

The titles and abstracts of a total of 1374 papers were screened for relevance, and duplicates were excluded. In the next step, studies were included if the content of the abstract included WHPP intervention(s). There were no restrictions regarding the year in which the study was published, the type of research, or the type of health promotion programme. Studies involving clinical populations were excluded. Our search yielded 58 research articles, the full texts of which were examined to see if the study mentioned the participation rate or data from which we could calculate the WHPP participation rate. A total of 37 articles met this condition. The elimination process according to the PRISMA model is presented in Figure 1.

### 2.3. Data Extraction and Analysis

In our scoping review, the missing participation rates were calculated as the number of employees participating in the WHPP divided by the number of employees initially invited, multiplied by 100. One third of the articles were independently reviewed by both authors to ensure the accuracy of the extracted data. The articles were then assessed for the level of employee participation, the type of programme or intervention, the target group(s) of the intervention, the type of business where the intervention took place, the strategies used to recruit or activate employees to participate in the intervention, and the study characteristics. Table 1 provides a summary of this analysis.

## 3. Results

### 3.1. Frequency of Reporting on Participation Rates

Only a small number of articles on WHPPs were found to report the participation rate. However, the number of articles reporting on participation rates was found to have increased in recent years. Of the 58 relevant articles found in selected bibliographic databases, only 37 studies (64%) reported participation rates. In terms of the year of publication, seven articles were published before 2000, and seven articles were published between 2000 and 2010. Since 2010, the number of articles has increased significantly with 10 or more in each subsequent 5-year period. The oldest study dates from 1988, and the most recent study dates from 2020.

Participation rates were found to be reported in different ways. A total of 23 studies reported the average or overall participation rate in health promotion programmes. Overall participation rates were reported for the specific programme as a whole, whereby the average participation rates were calculated across different interventions or groups. For the remaining 13 studies, average participation rates were calculated by the authors from individual participation rates associated with participant groups or interventions. However, the studies by Ott-Holland, Shepherd, and Ryan (2019) [28] and Brill et al., (1991) [29] differed substantially as they spanned three years between 2010 and 2012, with participation rates for the second and third years recorded in three time periods per year.

### 3.2. WHPP Participation Rates

Reported participation rates range from 3% to 100%, with over half of all studies reporting the average (or in some cases the overall) participation rate of less than 50% (20 studies). Two studies reported participation rates of less than 10% [20,30], five studies reported participation rates from 20% to 30% [31,32,33,34,35], five studies reported participation rates between 30% and 40% [21,29,36,37,38], and eight studies reported participation rates between 40% and 50% [22,23,24,39,40,41,42,43]. Four studies reported participation rates between 50% and 60% [25,44,45,46], five studies reported participation rates between 60% and 70% [47,48,49,50,51], four studies reported participation rates between 70% and 80% [26,27,52,53], two reported participation rates between 80% and 90% [12,54], and finally one study reported a participation rate between 90% and 100% [55].

For the programmes with the highest participation rates, we found no obvious correlation with company size, i.e., the number of employees involved. What is typical of these programmes is that employees were either enrolled or they volunteered. One of such programmes also offered a monetary prize/incentive [27]. A closer look at the programmes with the lowest participation rates showed that these were predominantly dealing with very large populations, however the strategies used to recruit or activate employees were not described. In one of the studies, a financial incentive was offered [20]. We also note that in the studies on WHPPs with low participation rates, the data relevant to understanding participation are more deficient than in the studies with higher participation rates, at least in terms of describing the process of recruiting employees. Lower participation rates appear more often in physical activity interventions than in those related to counselling and training.

### 3.3. Ways of Promoting Employee Involvement in WHPPs

Financial incentives are the most common form of encouraging participation in WHPPs, but do not seem to guarantee higher participation. Financial incentives were reported in nine studies. Eight articles reported voluntary participation in the programme and two reported assigning employees to a specific programme. Seventeen studies did not report how employees joined the programme or how participation was encouraged. Incentives and their relation to participation rates are difficult to compare directly, since various incentive sizes and incentive schemes were applied—some with fixed incentives, some with variable incentives, and some with stepped incentives. When examining the used financial incentives and the respective response rates (see Table 2), it appears that higher (financial) incentives tend to be associated with higher participation rates, although financial incentives do not guarantee higher participation rates in WHHPs.

### 3.4. Research Design and Duration

There is an apparent diversity in both the duration of the WHPP and the research design used. Only 13 studies reported the duration of the programme/intervention. The shortest study lasted three weeks [32] and the longest four years [38]. Some used qualitative or quantitative approaches, while others used only cross-sectional designs or questionnaires.

### 3.5. Types of WHPPs/Interventions

The types of interventions were found to vary widely, ranging from specific to combined interventions. Analysis revealed 17 WHPPs which specified only the general area of intervention, and 20 WHPPs which explicitly described the interventions, such as physical activity programmes that included stretching, exercise programmes, gym availability, and standard walking [28,29,42,55]; health education programmes included guidelines on fruit and vegetable consumption or nutrition in general, biometric screening tests and health risk assessments [27,36]. Health and life coaches were also available. One WHPP even offered free flu vaccinations [35]. Online programmes focusing on wellness and health or general self-care were also offered, including programmes on diabetes, depression, stress management, insomnia, back pain management, relaxation techniques, weight management, and binge eating [38,53]. Five WHPPs also offered smoking cessation interventions [35,38,42,52,54]. Some WHPPs were organised in the form of small group lectures, others offered cross-site activities, and some also included individual counselling [52]. Many WHPPs offered more than one intervention; whereby in some cases, employees were free to choose the intervention in which they wished to participate, and in others they were assigned a specific intervention.

### 3.6. Company Branches and Employee Positions

The analysis revealed that limited information was reported about the industry and even less about the position of participating employees, but a wide variety of work settings was reported. A total of 12 articles contained no information at all about the company, while 18 articles reported only on the industry or area in which the company operated. Moreover, as only 11 studies were found to report on the specific position of employees within the company, we did not include this factor in the additional analysis.

**Table 2:** WHPP study characteristics.

Author (Publication Year)	Type of Research			Type of WHPP Intervention	Participants/Target Group	Onboarding	Participation Rate (%)	
	Type of Research	Research Process	Time		Participant Characteristics		Overall	Divided Groups
T. Braun, C. Bambra, M. Booth, et al., (2015) [31]	An evaluation of the Bette Health at Work Award WHPP	Baseline and follow-up data on sickness-absence rates and programme costs collected via a web survey of all participating organisations. Changes over time were calculated using 95% confidence intervals of the mean, supplemented by hypothesis testing using a *t*-test.		Basic WHPP intervention with combined work environment changes and lifestyle interventions	232 participating workplaces; 209,319 employees		Response rate for full data 27%	Regional workforce 21.4%;
S.J.W. Robroek, S. Polinder, et al., (2012) [47]	A cluster randomised controlled trial, with departments within companies as the unit.	The intervention was compared with a standard programme consisting of a physical health check with face-to-face advice and personal feedback on a website.	2-years	Several website functionalities: action-oriented feedback, self-monitoring, possibility to ask questions, and monthly e-mail messages. Primary outcomes were meeting the guidelines for PA and fruit and vegetable intake.	Out of 924 employees, response was 666 at 12-month and 558 at 24-month follow-up	Participants enrolled in the trial; all participants gave written informed consent.	Calculated by author: 66%	1st year—72%; 2nd year—60%
R. Bourbonnais (2006) [48]	A quasi-experimental before-and-after design with a control group.	The pre-intervention measurement (M0) was conducted between February and April 2000 in the experimental and control hospitals by telephone interview. The first measurement after the intervention (Ml) took place in spring 2002.		Basic WHPP intervention	Men (138), women (536), age (18–45<); the study population is composed of nursing staff from the experimental and control hospitals, both of which provide general and specialised short-term care.		Calculated by author: 69.5%	M0, 73%—experimental hospital, 69%—control hospital; M1, 77%—experimental hospital, 62%—control hospital
M.A.J. Niessen, R.A. Kraaijenhagen, et al., (2012) [37]	Prevention Compass—a computerised knowledge-based reasoning system			Basic WHPP intervention	Employees employed at a Dutch financial service company during the January 2007 and July 2009 (11,252 invited employees).		3826 employees—34%	
R. Tsai, T. Alterman, et al., (2019) [45]	Cross-sectional study; the National Centre for Health Statistics National Health interview survey	A face-to-face household survey of a national cross-sectional sample; the 2 main outcomes of interest were availability of and participation in WHPPs.		Basic WHPP intervention	17,469 workers, of whom 8139 (46.6%) responded (18 years old<); participation outcome included only workers (n = 8131) who indicated that WHPPs were available.		Out of 46.6%, 4.744 (57.8%) participated	The occupations with the highest participation were arts, design, entertainment, sports and media (68.4%), management (68%), and community and social services (66.7%). The occupations with the lowest participation were agriculture, fishing and forestry (26%), food preparation and serving (42.4%), and construction and mining (45.3%).
M.M. Chen, A. C. Tsai, et al., (2016) [34]	A quasi-experiment; participation in health promotion activities	After group assignment, the 24-week intervention trial was divided into two phases. Phase I (4 weeks) focused on assigning workers to action groups, individual behaviour change planning, and refreshing workers’ health knowledge; phase II (follow-up 20 weeks) focused on implementing the planned lifestyle improvements.	4 weeks	Basic WHPP intervention	3 work sites; 1245 workers; 438 (35.2%) met the criteria; 264 showed interest; 108 participated	Participants signed a consent form for the study.	108 participated (=24.7%)—8alculate by author	
J. W. Grosch, T. Alterman, et al., (1998) [42]	A national cross-sectional probability sample of the U.S. civilian population. HP activities/programs; National Health Interview Survey	Data analysed from the 1994 NHIS; Interview		Training facilities, training programmes, screening tests, smoking cessation, health education programmes.	Out of 116,179 people, 19,738 were surveyed; 5219 people met the final criteria. Based on the weighted data, about 54% of the employees were male, 82% were classified as white, 13% as black, and 5% belonged to other ethnic groups (Asian, Hispanic, etc.). The average age was 39.2 years and the average education level was 13.8 years.		Overall 49.6%	From 32% to 5% in individual programs
S. Basu, M. Kiernan (2016) [20]	A first-generation, stochastic microsimulation model	Stochastic microsimulation model was applied to address the question of what level of incentive should be offered, and among which employees for any given type of incentive program-physical activity orientated		3000 worksite physical activity programs; worksite population—individual characteristics (demographics, health behaviours, health risks).	58,858 employees form 3000 firms with at least 50 employees—2005 to 2010	Average annual incentive of USD 143 per employee	9.70%	
K. McCleary, R. Z. Goetzel, et al., (2017) [32]	Analysed data from two independent surveys of employers (N = 1500) and the general population (N = 4611)	20-min online and telephone survey.	August 14—3 September 2015	Basic WHPP intervention	Employers: targeted primary decision makers regarding employee benefits at 1500 firms in the U.S., primarily small and mid-sized businesses (50 to 499 employees); n = 705. Employees: adults 18 to 64 years old living in the U.S. n = 1833.		Employees who participated in a WHPP (24.6%)	
C. J. Ott-Holland, W. J. Sheperd, et al., (2019) [28]	Cross-lagged SEM models using multisource data that control for prior levels of attitudes and behaviours.	Examination how wellness program participation levels relate to job satisfaction, performance, intention to stay and actual turnover over a 3-year period.	2010–2012	Basic WHPP intervention	Employees at branches of a U.S. financial institution based primarily in the Midwest; total number = 17,245 employees	Employees could earn up to USD 200 in health care premium deductions by participating. The deduction amount was determined by the number of points accumulated (USD 1 per point, 200-point maximum).		59.4% showed repeat participation in Y3
R. J. Mitchell, R. J. Ozminkowski, et al., (2013) [30]	Propensity score weighting and multiple regression			Care advocates, health coaches, and nurses delivered wellness, lifestyle coaching, disease management, and decision support services to employees with one or more identified health improvement opportunities.	Individuals employed by Optum clients between May 2010 and April 2011; 18–70 years; have insurance; participated in all types of programs, N = 131,011; 3793 participants, 127,218 non-participants.		3%	3%, 11%
R. P. Sloan, J. P. Gruman (1988) [40]	Questionnaire	Participants were asked to fill out the questionnaire during the wellness orientation session and to return it at the session’s end.		Basic WHPP intervention	192 subjects (129 female, 63 male) employees of AT&T Communications. Mean age was 35.81 (SD = 9.66) years, mean tenure was 9.49 (SD = 8.02) years.		48.40%	
J. L. M. Lindo, J. LaGrenade, et al., (2017) [49]	A descriptive cross-sectional study	An evaluation of workers socio-demographic, health status, and lifestyle data.		Basic WHPP intervention	385,500 employees in Kingston metropolitan in total; 1087 employees from large companies within a 10 km radius of downtown Kingston; 1020 individuals employed by two government ministries, three private-sector companies, and a quasi-government agency from 6 companies		Overall 62.9%	Varying 56–72.9%; lowest 49.22%, highest 80.4%; 1P-68.65%, 2P-57.27%, 3Q-80.2%, 4G-77.47%, 5G-49.22%, 6P-84.6%
F. Moy, A. A. B. Sallam, et al., (2006) [54]	A pre-test-post-test quasi-experimental study	Self-administered questionnaires were used to gather information on sociodemographic characteristics, medical history, and self-reported lifestyle behaviours. Interviews and focus group discussions were conducted after the survey to better understand some of the behaviours or perceptions. Anthropometric measurements, blood pressure, and biochemical measurements were taken at baseline and at 6-month intervals for 2 years.	2-year follow-up	Nutrition, occupational exposure, smoking	Number of eligible participants was 111 and 99 for the intervention and comparison groups; of these, 102 staff from the intervention and 84 staff from the comparison groups participated in the baseline health check.	Participation was voluntary and informed consent was given by all the participants.	Calculated by author: 88.35%	91.9% intervention group and 84.8% comparison group
J. Hoert, A. M. Herd, et al., (2018) [35]	A cross-sectional survey design—the tailored design method	The research examined the relationships between managerial support for health promotion, participation in wellness programmes, work stress, and positive health behaviours.		Participation in wellness activities, workplace stress, and health behaviours were measured. The public university and bank offered biometric screenings, on-site flu shots, on-site fitness facilities, and classes offered during the workday, health insurance take-up incentives, and wellness coaching services. The wholesaler’s wellness programme included biometric screenings, free flu shots, a diabetes prevention programme, coaching for blood pressure, diabetes, weight management, exercise, proper nutrition, and smoking cessation. Biggest Loser and Maintain Not Gain competitions, and provision of healthy snacks and water. The private university’s wellness programme included biometric screenings, free Zumba and yoga classes, a free lunch when an employee goes for a walk before or after lunch, and health-related “Lunch and Learn” sessions with information and discounts.	4 worksites—bank, private university, wholesale supplier, and public university, in U.S.; bank (n = 1058), private uni. (n = 197), wholesale supplier (n = 247), pub. N = 6500; 618 employees participated. Respondents were mostly full-time (89%), female (62%) and white (83%), and varied in age (14% were 30 years or younger and 36% were 51 years or older), education level (31% without a university degree and 23% with a university degree), length of employment (37% worked 5 years or fewer and 22% 16 years or more), and job function (20% in administration/clerical, 23% as supervisor/manager and 21% in professional).		Calculated by author: 21.25%	28% bank, 34% private uni., 20% wh. Supplier, 3% public uni.
G. Sorensen, A. Stoddard, et al., (1996) [51]	A randomised controlled study of an integrated health promotion/health protection intervention	A randomised, paired research design with the workplace as the unit of allocation examined the effectiveness of health promotion interventions targeting diet and smoking in 57 paired workplaces with self-administered survey.		Basic WHPP intervention	24 worksites in eastern and central Massachusetts; 160 eligible and invited worksites, 24 (15%) agreed to participate. Total of 4465 employees at the 12 worksites.	Worksites were randomly assigned to intervention and control conditions.	Worksite overall—62% (n = 2767)	Response rate range across worksites = 43–88%; program activities: nutrition—49.0%, occ. exposure—39.4%, smoking—34.2%
J. L. Hall, K. M. Kelly, et al., (2017) [41]	The Real Iowans Health Survey (RIHS)	A telephone survey of employed Iowans registered to vote.		Basic WHPP intervention	3396 voters contacted, 1603 completed the survey (47.2%); final sample: n = 1171 Iowans registered to vote, ages 18 to 65.		47.2% response rate	
B. W. Sherman, C. Addy (2017) [27]	Cross-sectional analysis of employee eligibility file and health benefits (wellness and claims) data	The data was analysed as part of a broader study examining health care utilisation and cost patterns. Employees were separated into 5 groups based on wage status. Data from self-insured employers participating in the RightOpt private exchange (Conduent HR Services) during 2014.	During 2014	An opportunity to participate in both HRA and biometric screening.	Active employees who were continuously enrolled in health insurance through the RightOpt private exchange in 2014 and for whom company-provided wage data was available. 42,936 active employees met the criteria for participation in the study. The mean age of the sample population is 43.5 years (SD, 11.2 years); 47% of the population is female.	An employer-specific incentive of USD 400 (1 employer) or USD 600 (3 employers) was offered to all enrolees for participating in the HRA and biometric screening.	The mean participation rate was 81.7% and 77.8% for completing the HRA and biometric screening. Calculated by the author: 79.75%	87% and 90% high wage 60% and 67% low wage;
S. P. Singleton, J. T. Fitzgerald, et al., (1993) [44]	A survey	A questionnaire was used to determine the desire for and willingness to participate in a campus wellness program.		Basic WHPP intervention	4.300 employees at Wayne State University; 2.401 responded; mean 41 y. o., 57% female, 43% male; 70% white, 22% black, 7% Asian; 44% earned between USD 20,000–40,000, 24% earned low income < USD 20,000, 32% earned high income > USD 40,000.		2.401—56% response rate	
R. J. Lewis, W. W. Huebner, et al., (1996) [36]	A descriptive study design	In a petrochemical research and development company where employees were offered a range of on-site wellness programmes that targeted the behavioural risks measured by the HRA. Demographic and behavioural risk characteristics of participants and non-participants were observed.		Health risk appraisal, wellness program, fitness centre	All employees (n = 2290) working continuously from 1 May 1990, through 28 February 1992. Most eligible employees were between 21 and 60 years of age, male, white, and had at least a post-high school education.		Calculated by author: 37%	Health Risk Appraisal—37% (843), Wellness Program—64% (1471), Fitness Centre—10% (151)
A. Smith-McLallen, D. Heller, et al., (2017) [46]	A cluster-randomised trial	Baseline measurements taken approximately 2 weeks before the intervention and follow-up measurements taken at 3, 6, and 9 months after baseline. Six employer groups were randomly selected and randomly assigned to condition.	9 months	A standard walking, enhanced program that included incentives, feedback, competitive challenges, and monthly wellness workshops.	474 employees from six employer groups; 234 in the enhanced condition and 225 in the standard condition; 19–77 y. o., 56% female.		Calculated by author: 53%	Calculated by author: enhanced group 58.25%, standard group 47.75%
B. Joslin, J. B. Lowe, et al., (2006) [39]	The cross-sectional design, following individuals’ decision to participate in a worksite wellness programme.	The Short Form-36 questionnaire designed specifically with the intention of measuring health related QOL. Survey packets were mailed to the study sample. One-week and three-week follow-ups were conducted in attempt to increase the survey response rate.		Basic WHPP intervention	Government employees in a midwestern United States community; 511 individuals employed by the county; 203 were wellness programme participants; 329 employees recruited. The mean age of survey respondents was 44 years.		N = 145 surveys; 44% response rate	58.5% within wellness participant group
B. M. Murphy, J. A. Schoenman, et al., (2010) [22]	Case studies of eight insurers	Telephone interviews with 20 informants.	November 2007—February 2008.	Wellness activities conducted at workplaces, on-site cafeterias and fitness facilities. At home telephone coaching and online classes were offered. Employer-subsidised fitness facilities.	20 informants	Cash rewards or gift certificates for completing programme components, with the HRA being the most commonly rewarded component. Cash rewards ranged from USD 25 to USD 400. Paid days off were also popular. A shift from activity-based to outcome-based incentives, with rewards tied to changing health risks and improving outcomes rather than just programme participation.	Calculated by author: 48.8%	From 8% with an external programme to 90% with an internal programme.
E. Largo-Wight, W. W. Chen, et al., (2011) [33]	Cross-sectional study	Web-based survey with 16-item workplace environment questionnaire was used (Nature Contact Questionnaire), to measure nature of contacts at work. An e-mail invitation along with the Web link was sent.		Basic WHPP intervention	A census of office staff at a southeastern university (n = 1622); mostly deskbound office staff (secretaries and office clerks); majority of the participants were women (92.9%) and white (82.5%), mean 42 y. o. -> n = 503 participated.	Participation was anonymous and voluntary.	The response rate was about 30% (n = 503).	
S. B. Gingerich, D. R. Anderson, et al., (2012) [26]	Retrospective cohort study conducted to observe the relationship between financial incentives and behaviour change programme registration, completion, and risk improvement rates.	Average registration rates, program completion rates, and risk improvement rates were compared using t-tests for companies that did versus did not offer incentives. Correlations between incentive value and outcome variables were assessed using Pearson correlations.		Behaviour change program. Financial incentives offered for completion of a behaviour change program as part of a WHHP.	24 organisations (n = 511,060 eligible employees) that offered comprehensive worksite health promotion programmes.	Incentive values ranged from USD 0 to USD 360.	Calculated by author: 79.7%	With initiative 82.9%, without initiative 76.4%
L. C. Williams, B. T. Day (2011) [38]	Quasi-experimental, pre-post, treatment-comparison design	Outcomes were calculated using health insurance enrolment and claims history. Participating workers were compared with non-participants using generalised linear mixed models to examine changes in costs and claims. The variables of interest were participation rates, expenditure on medical, occupational and pharmaceutical services, inpatient admissions, emergency room visits, and use of preventive services.	4 years	Interventions for clinical assessment and education included web-based programmes to manage diabetes, depression, back pain or general self-care, on-site biometric screening, personalised nutritional counselling, and group classes to prevent and manage heart disease, diabetes, osteoporosis or depression. Nutrition and weight management interventions included web-based programmes for nutrition, weight management or binge eating, group courses to teach healthy eating habits, and a newsletter campaign on nutrition. Stress management interventions included web-based programmes for insomnia and relaxation, and group courses to teach relaxation. Smoking cessation was promoted through web-based, individual, and group interventions.	Of the 643 employers who participated in all study years, 398 were active in the programme for at least 3 years between 2004 and 2007. The 47 employers with a participation rate of 25% or more over 2 plus years and an average participation rate of 20% or more were classified as engaged.		Calculated by author: 33.7%	Median participation rate: 2004—11.4%, 2005—39.5% 2006—40.7%, 2007—43.2%
L. Linnan, D. F. Tate, et al., (2012) [56]	The WAY to Health study was a group-randomised three-arm weight loss trial.	The weight loss trial in which overweight and obese employees were nested within each university, with the university being the unit of randomisation. Each enrolled community college assigned a contact that was invited to participate in a WAY to Health kick-off event to be informed about the purpose and timeline of the study.		Weight loss study	1200 employees from all colleges were enrolled. Employees who met eligibility requirements were recruited. Eligible employees aged at least 18 years, were employed in selected organisations and had a body mass index (BMI) greater than 25 kg/m^2^.	Enrolled, medical consent required.	Acceptance rate: 89%	Participation rate of 70.3% among the 354 employees who required medical consent.
M. K. Hunt, E. M. Barbeau, et al., (2007) [52]	A process evaluation from Healthy Directions–Small Business (HD-SB); one of two randomised, controlled intervention studies that were part of the Harvard Cancer Prevention Program Project.	Study aimed to reduce cancer risk among multi-ethnic workers in small manufacturing businesses by increasing fruit and vegetable consumption, physical activity, daily multivitamin intake, and decreasing consumption of red meat.		At the individual/interpersonal level, it provided one-to-one, small group, and worksite-wide activities: quitting smoking, healthy eating or nutrition, workplace health and safety, physical activity or exercise. Examples of worksite-wide activities included a physical activity contest, a fruit and vegetable challenge, and large displays that were tailored to each worksite.	A total of 131 companies met the study eligibility criteria; of these, 26 agreed to participate; of 26 worksites, follow-up was completed on 24 sites. The majority were men, approximately half of the workers had no more than a high school education, and the median age range was 35 to 50 years. Thirteen sites were randomised to the intervention condition and 13 to the minimal-intervention control condition.	Recruited—worksite representative agreed to be randomly assigned to the intervention condition.	The survey response rate at the final time was 77%	Of physical activity and exercise programs, 19% participation rate for NHIS and HD-SB had 54.4% participation rate. A total of 28% of NHIS respondents and 62% of HD-SB participated in nutrition education. 50.4% of NHIS respondents and 54% of HD-SB workers reported participation in workplace health and safety. 6% of NHIS respondents and 25% of HD-SB workers reported participating in smoking cessation programs.
R. M. Gartley, J. L. Prosser (2011) [55]	A nonrandomised, descriptive, pre-post intervention design	Conducted at two industrial sites: a beverage manufacturer and a tin factory with manual workers. Both factories have on-site occupational health clinics where each investigator in the study is employed. Aim: to determine the effects of a pre-shift stretching programme on work-related musculoskeletal injuries and to assess daily participation in the programme during the day. Data were collected and compared with injury events during the same period 1 year earlier.		Stretching programme—the programme consisted of nine stretching exercises for the neck, upper and lower back, hamstrings, shoulders, quadriceps, arms, and ankles.	37 warehouse loaders and 18 delivery drivers engaged in manual labour; 47% of study participants were warehouse personnel, 23% were delivery drivers, and 30% were tin mill laborers. 78 male, 1 female workers; average age 50.4 years; participants in the exercise program were excluded if they incurred a work-related musculoskeletal injury during the study period.	Volunteer workers from two separate companies.	Attendance sheets indicated 100% participation and 100% program completion.	
E. Haisley, K. G. Volpp, et al., (2012) [24]	Logistic regression analysis. A two-arm, randomised, controlled trial with a convenience comparison sample.	Employees were assigned to one of three arms. Assignment to a treatment arm versus the nontreatment arm was determined by management. Assignment to an arm among those eligible for treatment was randomised by office. The main dependent measure in the study was HRA completion rate.	4 weeks	One reminder e-mail was sent each week for 4 weeks.	The study was conducted in a health management company in all 14 branches spread across the country with 15 or more employees, resulting in 1299 eligible employees. The sample was predominantly female (85%), with an average age of 41 years and an average length of service of 2.86 years. The median household income, estimated based on the median income for the employee’s postcode, was USD 43,084.	All employees were eligible to receive USD 25 for completing the HRA. Those in the lottery condition were assigned to teams of four to eight people and, conditional on HRA completion, were entered into a lottery with a prize of USD 100 (expected value, USD 25) and a bonus value of an additional USD 25 if 80% of team members participated. Those in the grocery gift certificate condition who completed an HRA received a USD 25 grocery gift certificate. Those in the comparison condition received no additional incentive.	Calculated by author: 49.33%	The HRA completion rate was 64% in the lottery group (n = 489), 44% in the grocery voucher group (n = 184), and 40% in the comparison group (n = 626).
P. A. Brill, H. W. Kohl, et al., (1991) [29]	A pilot programme offered to teachers in four schools.	The recruitment, retention, and success of a workplace health promotion programme was studied among different demographic groups.		Employees underwent a health screening consisting of an assessment of health habits, measurement of clinical variables, a physical fitness test and a medical examination. Organised activity classes and health education classes.	Employees (n = 11,830) of Dallas, Texas Independent School District. Enrolled employees (n = 3873)	A participation fee of USD 120 per participating employee.	Calculated by author: 32.7%	
E. L. D. Seaverson, J. Grossmeier, et al., (2009) [23]	A cross-sectional study design to examine factors that influence employee participation, including incentive value, incentive design, communications strategy, and worksite culture.	The study considered participation across a group of select organisations that provided financial incentives to promote HRA participation as part of a comprehensive worksite health promotion strategy. It examined the relationships between the use of financial incentives, extensive communication or a strong company culture and participation rates.	A single program year occurring during 2004–2006.	The primary outcome of interest was participation in a StayWell HRA. These assessments include a variety of questions on topics such as demographics, chronic conditions, health status, and health behaviours.	The sample consisted of 36 employers (n = 559,988 employees) representing primarily large companies across a broad range of private and public sector industries, including manufacturing, service, finance and insurance, retail, and utility.	Most organisations used cash-based incentives (44%) or incentives integrated into the health plan (44%), and the remainder used non-financial incentives of low value (USD 25). The mean value of incentives was just over USD 100, and most organisations offered incentives worth between USD 50 and USD 100.	49% (n = 275,100) participated in the HRA	Non-financial incenitives (n = 4): communications strategy—27–44%, worksite culture 33–41%; cash incentives (n = 16): C.S.—33–51%, W.C.—37–51%; benefits-integrated Incentives (n = 16): C.S.—41–65%, W.C.—53–69%
I. Lowensteyn, V. Berberian, et al., (2019) [53]	Prospective cohort study with a within-participant pre-post design.	The participation rate and observed health outcomes were evaluated: weight, waist circumference, blood pressure, total and high-density lipoprotein (HDL) cholesterol, and glycated haemoglobin.		Web-based challenges (team or individual) incorporating gamification strategies to improve exercise, nutrition, weight reduction, and mental health management behaviours. The customised wellness program was delivered using a gradual introduction of the key elements including MOVE IT (exercise), FUEL IT (nutrition and weight management), and BALANCE IT (mental health).	All permanent employees (n = 775) of a national company located in Canada were eligible to participate.	Participation was voluntary and free of charge.	Participation rates in the health screenings were 78% (baseline), 54% (year 1), and 56% (year 2). At baseline, 74% of 21alculat participated in the biometric screening; 21alculate by author: 76%	Participation in the biometric screening included 571 (78%) of the 735 eligible employees at baseline (319 head office and 252 field), 396 (54%) at year 1 (208 head office and 188 field), and 409 (56%) at year 2 (231 head office and 178 field). There were 314 (43%) employees who attended both the baseline and first-year screening, 310 (42%) who attended both the baseline and second- year screening, and 189 (26%) employees who attended all 3 screenings.
B. Batorsky, E. Taylor, et al., (2016) [25]	A cross-sectional analysis of nationally representative survey data combined with an administrative business database.	Logistic regressions of incentive type on employer characteristics were used to determine what types of employers are more likely to offer which type of incentives. A generalised linear model of participation rate was used to determine the relationship between incentive type and participation. The research design is a cross-sectional analysis based on two data sets: The RAND employer survey (RAND survey) and the Dun and Bradstreet (DandB) employer database.		Basic WHPP intervention	Random sampling of U.S. companies within strata based on industry and number of employees was used to determine a final sample of 3000 companies. Of these, 19% returned completed surveys. Of the 3000 employers in the final sample, 589 (19%) returned completed surveys.	Of the employers who responded to the survey, 69% offer a wellness programme. Of those that offer a wellness programme, 68% use financial incentives to encourage participation. More than a third of these programmes (39%) use cash incentives with a maximum annual value of more than USD 100. In 14% of the programmes that offer incentives, sanctions are used in addition to rewards.	Calculated by author: 60%	Penalty and high-value incentives were associated with participation rates of 68% and 52%
S. S. Bull, C. Gillette, et al., (2003) [50]	The RE-AIM (Reach, Efficacy–Adoption, Implementation, Maintenance) evaluation	Using the RE-AIM framework, they summarise the characteristics and findings of selected studies to document the reporting of the intervention’s reach, adoption, implementation, and maintenance.		Basic WHPP intervention	The authors reviewed a total of 24 publications from 11 leading journals on the topic of health behaviour.		The median adoption participation rate was 56.5%.	The range of participation rates among eligible workers varied across the studies—from 8% (34) to 97%. Participation rates at the individual worker level: average participation rate of 63.5% across studies.

The authors’ own calculation of the participation rate in the WHPP.

## 4. Discussion

The conclusions that emerge from our analysis represent a valuable contribution to several aspects of the WHPP research (paragraphs one and two) and practice (paragraph three).

(1) The question of redirecting the research focus in WHPPs should be raised, as programme organisers/facilitators (sponsors, coaches) waste valuable resources on programmes of limited general impact, given that the benefits to the target population and those who would need the interventions most may be (very) small. Considering that the scoping review showed that the focus of WHPP research avoids the burning issue of low participation rates, it is not surprising that the incidence of modern work-related diseases (obesity, hypertension, burnout, etc.) continues to rise despite the abundance of programmes and funding. This raises the broader question of how the effects and impacts of WHPP interventions introduced can be considered positive when only a (relatively) small proportion of workers participate in such programmes. At the very least, the effectiveness and efficiency of such programmes are questionable, as many resources are spent on (very) limited effects, which more often than not benefit those who are already health conscious.
(2) Reporting on participation rates in WHPPs should be made more consistent and comparable. In particular, it would be useful to report participation rates for each type of intervention (physical activity, workshops, active breaks, awareness programmes, etc.) by target group or type of incentive, and to summarise participation rates for composite interventions. In short, it would be useful to monitor participation rates by at least the most important factors so as to obtain comparable data on uptake. We also believe that an in-depth meta-analysis focusing on the determinants of participation in WHPPs would be imperative to understand the reasons for such low participation rates.
(3) Not to overreach the aim of this study, we need to highlight an issue that is often left unaddressed. While we found that participation rates were reported in a few studies, in the vast majority of articles on WHPP they were not mentioned. Our scoping review thus draws attention to the potential problem of scientific misconduct [57], more specifically a type of reporting bias: “a systematic distortion that arises from the selective disclosure or withholding of information by parties involved in the design, conduct, analysis, or dissemination of a study or research findings” called publication bias [58]. Publication bias occurs when the research findings influence the decision to publish or otherwise disseminate them [59].

Chan and Altman (2005) [60] list many different reasons why research results go unreported. Researchers seem to make the decision to omit certain results for a combination of reasons, such as space limitations in journals, significance of the results, and specific statistical results. To this we could add the pressure of having to publish. We might hypothesise that in the world of academic research with the prevailing “publish or perish” culture, studies with higher participation rates are more likely to be published than those with “disturbingly low” participation rates despite the similar quality of conduct and design. On the other hand, authors might refrain from reporting participation rates if they appear worryingly low in the hope that this issue will not be raised during the review process. Whatever the reason, one possible consequence is that the balance of outcomes tips in favour of positive outcomes [59], which further distances the research focus from what needs to be addressed: low participation rates in WHPPs.

## 5. Limitations

The following limitations may have appeared in our scoping review. First, our literature search was limited to the selected electronic article databases, where there is considerable overlap in content. It is also possible that some useful studies were overlooked, as we only focused on English language publications in scientific journals. However, we assume this does not to have a significant impact on the results. Another potential limitation is that many interventions or studies had been conducted in the field, and the results of such studies were either not evaluated or not accurately recorded in the literature.

## 6. Conclusions

The main findings of the systematic review can be divided into three sections pertaining to the reporting of participation in the WHPP, the level of reported participation rates with the indication of trends in higher or lower participation rates, and the issue of comparability of reported rates. The proportion of appropriate studies relative to the baseline articles identified is relatively small, with research still primarily concerned with reporting the effects of interventions rather than focusing (specifically) on the circumstances of the uptake. In more than half of the studies reviewed, reported participation rates were relatively low—below 50%. There seems to be no obvious interconnection pattern between the size of the company, i.e., the number of employees involved, and reported participation rates. Higher (financial) incentives tend to be associated with higher participation rates; however, monetary incentives do not guarantee higher participation rates in WHHPs. Lower participation rates are more striking for physical activity interventions than for those related to counselling and training. The types of WHPPs vary, from specific to composite, as do the types of research designs used to measure their impact. A great deal of variability was also found in the delivery of the programmes. Reporting on the industry and workers’ position in the company was found to be very sparse. Most importantly, reported participation rates are tied either to different target groups or to the type of intervention or to single points in time in the research. As a result, it is difficult to establish uniform criteria for comparing reported participation rates, as they are difficult to compare directly. Nevertheless, the relationship between participation rates and intervention effectiveness should also be thoroughly explored.

Several implications emerge from these findings. Despite the importance of the WHPP design and its efficacy, research in the field of WHPPs should include analysing the mechanisms needed to improve WHPP participation rates, while reporting on WHPP participation rates should be made more consistent and comparable. An in-depth meta-analysis focusing on the determinants of participation in WHPPs would be imperative to shed light on the reasons for low participation rates. Although our research has only briefly touched on the (mezzo-level) determinants of participation, other possible micro-level (workers), mezzo-level (organisation, WHPPs), and macro-level (legislation, taxation, etc.) determinants should be thoroughly investigated. Second, the scientific community should closely examine the possibility of reporting bias in studies with low WHPP participation rates and, if identified, address it openly. Solutions to this problem are already known but do not appear to have been fully implemented, at least in the case of WHPPs. First, researchers and journal editors should ensure that complete data are made available for all studies, regardless of their values. In addition, as suggested by Richards and Onakpoya (2019) [58], post-study measures should be implemented. Reporting guidelines and other checklists and tools have been developed to assess the risk of reporting bias in studies, including the Cochrane risk of bias tool, GRADE, and ORBIT-II [61].

## Figures and Tables

**Table 1:** Financial incentives and corresponding participation rates.

Study (Publication Year)	Overall or Average Participation Rate (%)	Financial Incentive
Basu et al., (2016) [20]	9.7	USD 143
Hennrikus et al., (1996) [21]	39	USD 10
Murphy et al., (2010) [22]	48.8	From USD 25 to USD 400
Seaverson et al., (2009) [23]	49	From USD 50 to USD 100
Haisley et al., (2012) [24]	49.3	USD 25 with option for additional USD 100
Batorsky et al., (2016) [25]	60	≤USD 100
Gingerich et al., (2012) [26]	79.7	From USD 0 to USD 360
Sherman et al., (2018) [27]	79.8	From USD 400 to USD 600
Ott-Holland et al., (2019) [28]	n.a.	Up to USD 200

## Data Availability

Not applicable.

## References

[1] Ageing and Employment Policies—Statistics on Average Effective Age of Labour Market Exit—OECD. https://www.oecd.org/employment/emp/average-effective-age-of-labour-market-exit.htm.

[2] Neupane S., Kyrönlahti S., Oakman J., Siukola A., Riekhoff A.-J., Kuivalainen S., Nygård C.-H. (2022). Can Workplace Intervention Prolong Work Life of Older Workers? A Quasi-Experimental Study. Int. Arch. Occup. Environ. Health.

[3] Proper K.I., van Oostrom S.H. (2019). The Effectiveness of Workplace Health Promotion Interventions on Physical and Mental Health Outcomes—A Systematic Review of Reviews. Scand. J. Work. Environ. Health.

[4] Nielsen R.A., Midtsundstad T.I. (2021). Do Workplace Health-Promotion Interventions Targeting Employees with Poor Health Reduce Sick-Leave Probability and Disability Rates?. Scand. J. Public Health.

[5] Magnavita N. (2017). Productive Aging, Work Engagement and Participation of Older Workers. A Triadic Approach to Health and Safety in the Workplace. Epidemiol. Biostat. Public Health.

[6] Poscia A., Moscato U., La Milia D.I., Milovanovic S., Stojanovic J., Borghini A., Collamati A., Ricciardi W., Magnavita N. (2016). Workplace Health Promotion for Older Workers: A Systematic Literature Review. BMC Health Serv. Res..

[7] Kuoppala J., Lamminpää A., Husman P. (2008). Work Health Promotion, Job Well-Being, and Sickness Absences—A Systematic Review and Meta-Analysis. J. Occup. Environ. Med..

[8] O’Donnell M.P. (2015). What Is the ROI for Workplace Health Promotion? It Really Does Depend, and That’s the Point. Am. J. Health Promot. AJHP.

[9] Robroek S.J., van Lenthe F.J., van Empelen P., Burdorf A. (2009). Determinants of Participation in Worksite Health Promotion Programmes: A Systematic Review. Int. J. Behav. Nutr. Phys. Act..

[10] Lier L.M., Breuer C., Dallmeyer S. (2019). Organizational-Level Determinants of Participation in Workplace Health Promotion Programs: A Cross-Company Study. BMC Public Health.

[11] Nöhammer E., Schusterschitz C., Stummer H. (2010). Determinants of Employee Participation in Workplace Health Promotion. Int. J. Workplace Health Manag..

[12] Linnan L.A., Sorensen G., Colditz G., Klar N., Emmons K.M. (2001). Using Theory to Understand the Multiple Determinants of Low Participation in Worksite Health Promotion Programs. Health Educ. Behav..

[13] Rozman N., Širok K. (2020). E-Platform as an Innovative Approach to Workplace Health Promotion for Ageing Workforce. Eur. J. Public Health.

[14] Jordan S., von der Lippe E., Starker A., Hoebel J., Franke A. (2015). Einflussfaktoren Für Die Teilnahme an Bonusprogrammen Der Gesetzlichen Krankenversicherung. Ergebnisse Der Studie “Gesundheit in Deutschland Aktuell”. Gesundheitswesen.

[15] Baker C., Courtney P., Kubinakova K., Crone D., Billingham D. (2020). Assessing the Broader Social Outcomes of a Community Health Programme through a Social-Ecological Framework. Int. J. Health Promot. Educ..

[16] Rongen A., Robroek S.J., van Ginkel W., Lindeboom D., Altink B., Burdorf A. (2014). Barriers and Facilitators for Participation in Health Promotion Programs among Employees: A Six-Month Follow-up Study. BMC Public Health.

[17] Anderson D.R., Carter M., Rahrig Jenkins K., Karjalainen T., William Whitmer R. (2012). Toward an Employee Health Management Research Agenda—What Are the Research Priorities?. Am. J. Health Promot..

[18] Ryde G.C., Gilson N.D., Burton N.W., Brown W.J. (2013). Recruitment Rates in Workplace Physical Activity Interventions: Characteristics for Success. Am. J. Health Promot..

[19] Moher D., Liberati A., Tetzlaff J., Altman D.G., Group T.P. (2009). Preferred Reporting Items for Systematic Reviews and Meta-Analyses: The PRISMA Statement. PLOS Med..

[20] Basu S., Kiernan M. (2016). A Simulation Modeling Framework to Optimize Programs Using Financial Incentives to Motivate Health Behavior Change. Med. Decis. Mak..

[21] Hennrikus D.J., Jeffery R.W. (1996). Worksite Intervention for Weight Control: A Review of the Literature. Am. J. Health Promot..

[22] Murphy B.M., Schoenman J.A., Pirani H. (2010). Health Insurers Promoting Employee Wellness: Strategies, Program Components and Results. Am. J. Health Promot..

[23] Seaverson E.L.D., Grossmeier J., Miller T.M., Anderson D.R. (2009). The Role of Incentive Design, Incentive Value, Communications Strategy, and Worksite Culture on Health Risk Assessment Participation. Am. J. Health Promot..

[24] Haisley E., Volpp K.G., Pellathy T., Loewenstein G. (2012). The Impact of Alternative Incentive Schemes on Completion of Health Risk Assessments. Am. J. Health Promot..

[25] Batorsky B., Taylor E., Huang C., Liu H., Mattke S. (2016). Understanding the Relationship between Incentive Design and Participation in U.S. Workplace Wellness Programs. Am. J. Health Promot..

[26] Gingerich S.B., Anderson D.R., Koland H. (2012). Impact of Financial Incentives on Behavior Change Program Participation and Risk Reduction in Worksite Health Promotion. Am. J. Health Promot..

[27] Sherman B.W., Addy C. (2018). Association of Wage with Employee Participation in Health Assessments and Biometric Screening. Am. J. Health Promot..

[28] Ott-Holland C.J., Shepherd W.J., Ryan A.M. (2019). Examining Wellness Programs over Time: Predicting Participation and Workplace Outcomes. J. Occup. Health Psychol..

[29] Brill P.A., Kohl H.W., Rogers T., Collingwood T.R., Sterling C.L., Blair S.N. (1991). The Relationship between Sociodemographic Characteristics and Recruitment, Retention, and Health Improvements in a Worksite Health Promotion Program. Am. J. Health Promot..

[30] Mitchell R.J., Ozminkowski R.J., Serxner S. (2013). Improving Employee Productivity Through Improved Health. J. Occup. Environ. Med..

[31] Braun T., Bambra C., Booth M., Adetayo K., Milne E.M.G. (2015). Better Health at Work? An Evaluation of the Effects and Cost-Benefits of a Structured Workplace Health Improvement Programme in Reducing Sickness Absence. J. Public Health.

[32] McCleary K., Goetzel R.Z., Roemer E.C., Berko J., Kent K., Torre H.D.L. (2017). Employer and Employee Opinions About Workplace Health Promotion (Wellness) Programs: Results of the 2015 Harris Poll Nielsen Survey. J. Occup. Environ. Med..

[33] Largo-Wight E., Chen W.W., Dodd V., Weiler R. (2011). Healthy Workplaces: The Effects of Nature Contact at Work on Employee Stress and Health. Public Health Rep..

[34] Chen M.-M., Tsai A.C., Wang J.-Y. (2016). The Effectiveness and Barriers of Implementing a Workplace Health Promotion Program to Improve Metabolic Disorders in Older Workers in Taiwan. Glob. Health Promot..

[35] Hoert J., Herd A.M., Hambrick M. (2018). The Role of Leadership Support for Health Promotion in Employee Wellness Program Participation, Perceived Job Stress, and Health Behaviors. Am. J. Health Promot..

[36] Lewis R.J., Huebner W.W., Yarborough C.M. (1996). Characteristics of Participants and Nonparticipants in Worksite Health Promotion. Am. J. Health Promot..

[37] Niessen M.A.J., Kraaijenhagen R.A., Dijkgraaf M.G.W., Van Pelt D., Van Kalken C.K., Peek N. (2012). Impact of a Web-Based Worksite Health Promotion Program on Absenteeism. J. Occup. Environ. Med..

[38] Williams L.C., Day B.T. (2011). Medical Cost Savings for Web-Based Wellness Program Participants from Employers Engaged in Health Promotion Activities. Am. J. Health Promot..

[39] Joslin B., Lowe J.B., Peterson N.A. (2006). Employee Characteristics and Participation in a Worksite Wellness Programme. Health Educ. J..

[40] Sloan R.P., Gruman J.C. (1988). Participation in Workplace Health Promotion Programs: The Contribution of Health and Organizational Factors. Health Educ. Q..

[41] Hall J.L., Kelly K.M., Burmeister L.F., Merchant J.A. (2017). Workforce Characteristics and Attitudes Regarding Participation in Worksite Wellness Programs. Am. J. Health Promot..

[42] Grosch J.W., Alterman T., Petersen M.R., Murphy L.R. (1998). Worksite Health Promotion Programs in the U.S.: Factors Associated with Availability and Participation. Am. J. Health Promot..

[43] Grossmeier J., Castle P.H., Pitts J.S., Saringer C., Jenkins K.R., Imboden M.T., Mangen D.J., Johnson S.S., Noeldner S.P., Mason S.T. (2020). Workplace Well-Being Factors That Predict Employee Participation, Health and Medical Cost Impact, and Perceived Support. Am. J. Health Promot..

[44] Singleton S.P., Fitzgerald J.T., Engels H.-J., Wirth J.C. (1993). Attitudes of Employees for an On-Campus Health Promotion Program at a Large Urban University. Eval. Health Prof..

[45] Tsai R., Alterman T., Grosch J.W., Luckhaupt S.E. (2019). Availability of and Participation in Workplace Health Promotion Programs by Sociodemographic, Occupation, and Work Organization Characteristics in US Workers. Am. J. Health Promot..

[46] Smith-McLallen A., Heller D., Vernisi K., Gulick D., Cruz S., Snyder R.L. (2017). Comparative Effectiveness of Two Walking Interventions on Participation, Step Counts, and Health. Am. J. Health Promot..

[47] Robroek S.J.W., van de Vathorst S., Hilhorst M.T., Burdorf A. (2012). Moral Issues in Workplace Health Promotion. Int. Arch. Occup. Environ. Health.

[48] Bourbonnais R. (2006). Effectiveness of a Participative Intervention on Psychosocial Work Factors to Prevent Mental Health Problems in a Hospital Setting. Occup. Environ. Med..

[49] Lindo J.L.M., LaGrenade J., Eldemire-Shearer D. (2017). The Health of Office-Based Workers in Kingston, Jamaica. Workplace Health Saf..

[50] Bull S.S., Gillette C., Glasgow R.E., Estabrooks P. (2003). Work Site Health Promotion Research: To What Extent Can We Generalize the Results and What Is Needed to Translate Research to Practice?. Health Educ. Behav..

[51] Sorensen G., Stoddard A., Ockene J.K., Hunt M.K., Youngstrom R. (1996). Worker Participation in an Integrated Health Promotion/Health Protection Program: Results from the WellWorks Project. Health Educ. Q..

[52] Hunt M.K., Barbeau E.M., Lederman R., Stoddard A.M., Chetkovich C., Goldman R., Wallace L., Sorensen G. (2007). Process Evaluation Results from the Healthy Directions–Small Business Study. Health Educ. Behav..

[53] Lowensteyn I., Berberian V., Berger C., Da Costa D., Joseph L., Grover S.A. (2019). The Sustainability of a Workplace Wellness Program That Incorporates Gamification Principles: Participant Engagement and Health Benefits After 2 Years. Am. J. Health Promot..

[54] Moy F., Sallam A.A.B., Wong M. (2006). The Results of a Worksite Health Promotion Programme in Kuala Lumpur, Malaysia. Health Promot. Int..

[55] Gartley R.M., Prosser J.L. (2011). Stretching to Prevent Musculoskeletal Injuries. AAOHN J..

[56] Linnan L., Tate D.F., Harrington C.B., Brooks-Russell A., Finkelstein E., Bangdiwala S., Birken B., Britt A. (2012). Organizational- and Employee-Level Recruitment into a Worksite-Based Weight Loss Study. Clinical Trials.

[57] Fanelli D. (2009). How Many Scientists Fabricate and Falsify Research? A Systematic Review and Meta-Analysis of Survey Data. PLoS ONE.

[58] Richards C., Onakpoya I. (2019). Catalog of Bias. https://catalogofbias.org/biases/reporting-biases/.

[59] Song F., Parekh S., Hooper L., Loke Y.K., Ryder J., Sutton A.J., Hing C., Kwok C.S., Pang C., Harvey I. (2010). Dissemination and Publication of Research Findings: An Updated Review of Related Biases. Health Technol. Assess..

[60] Chan A.-W., Altman D.G. (2005). Identifying Outcome Reporting Bias in Randomised Trials on PubMed: Review of Publications and Survey of Authors. BMJ.

[61] Page M.J., McKenzie J.E., Bossuyt P.M., Boutron I., Hoffmann T.C., Mulrow C.D., Shamseer L., Tetzlaff J.M., Akl E.A., Brennan S.E. (2021). The PRISMA 2020 Statement: An Updated Guideline for Reporting Systematic Reviews. BMJ.

